# Impact of Low Blood Lead Concentrations on IQ and School Performance in Chinese Children

**DOI:** 10.1371/journal.pone.0065230

**Published:** 2013-05-29

**Authors:** Jianghong Liu, Linda Li, Yingjie Wang, Chonghuai Yan, Xianchen Liu

**Affiliations:** 1 University of Pennsylvania, School of Nursing and School of Medicine, Philadelphia, Pennsylvania, United States of America; 2 Xinhua Hospital, MOE-Shanghai Key Laboratory of Children's Environmental Health, Shanghai Jiaotong University School of Medicine, Shanghai, China; 3 Indiana University, School of Medicine, Indianapolis, Indiana, United States of America; 4 Shandong University, School of Public Health, Jinan, China; The Ohio State University, United States of America

## Abstract

**Objectives:**

Examine the relationships between blood lead concentrations and children's intelligence quotient (IQ) and school performance.

**Participants and Methods:**

Participants were 1341 children (738 boys and 603 girls) from Jintan, China. Blood lead concentrations were measured when children were 3–5 years old. IQ was assessed using the Chinese version and norms of the Wechsler Preschool and Primary Scale of Intelligence – Revised when children were 6 years old. School performance was assessed by standardized city tests on 3 major subjects (Chinese, Math, and English [as a foreign language]) when children were age 8–10 years.

**Results:**

Mean blood lead concentration was 6.43 µg/dL (SD = 2.64). For blood lead concentrations, 7.8% of children (*n* = 105) had ≥10.0 µg/dL, 13.8% (*n* = 185) had 8.0 to <10.0 µg/dL, and 78.4% (*n* = 1051) had <8.0 µg/dL. Compared to children with blood lead concentrations <8 µg/dL, those with blood lead concentrations ≥8 µg/dL scored 2–3 points lower in IQ and 5–6 points lower in school tests. There were no significant differences in IQ or school tests between children with blood lead concentrations groups 8–10 and ≥10 µg/dL. After adjustment for child and family characteristics and IQ, blood lead concentrations ≥10 µg/dL vs <8 µg/dL at ages 3–5 years was associated with reduced scores on school tests at age 8–10 years (Chinese, β = −3.54, 95%CI = −6.46, −0.63; Math, β = −4.63, 95%CI = −7.86, −1.40; English, β = −4.66, 95%CI = −8.09, −1.23). IQ partially mediated the relationship between elevated blood lead concentrations and later school performance.

**Conclusions:**

Findings support that blood lead concentrations in early childhood, even <10 µg/dL, have a long-term negative impact on cognitive development. The association between blood lead concentrations 8–10 µg/dL and cognitive development needs further study in Chinese children and children from other developing countries.

## Introduction

Childhood lead exposure is still an important public health problem in the world, predisposing children at risk of cognitive deficits and behavioral problems [1]–[3]. Emerging evidence has also suggested that even children with blood lead concentrations<10 µg/dL are at significant risk for reduced cognitive development and functioning, including intelligence quotient (IQ) deficits [4]–[10] and poor academic performance [11], [12]. Alarmingly, deficits in intellectual abilities and elevated risks for behavioral problems may persist into adolescence and even adulthood [13]–[17]. Despite increasing attention given to the importance of both elevated (≥10 µg/dL) and lower (<10 µg/dL) blood lead concentrations on children's cognitive development, however, several questions remain. Although previous studies have revealed that blood lead concentrations <10 µg/dL were related to poorer neurocognitive outcomes in children (e.g. [3], [5], [18]), studies in this area are still limited. Even more recently, the US Centers for Disease Control (CDC) eliminated the terminology “level of concern”. Children with elevated blood lead concentrations will instead be identified using a reference value based on the 97.5th percentile of the National Health and Nutrition Examination Survey (NHANES)-generated blood lead concentration distribution in children aged 1–5 years old; currently, this value is 5 µg/dl [19]. Since most studies have used cohorts from Western countries, it is unclear whether their findings are replicable in other developing countries, such as China, where lead concentrations and prevalence of lead exposure are much higher [3], [20]. In areas of high lead exposure, effects of lower concentrations of lead on children's developmental function may differ. In addition, the effects of lead on both IQ and school performance have rarely been examined together. Although Surkan et al. [10] demonstrated significantly reduced IQ and academic performance in 6–10 year old children, it is still unclear whether IQ deficits due to lead exposure subsequently results in poor academic achievement. Finally, regarding the effects of low lead exposure on the specific type of IQ (e.g., Performance IQ [PIQ]), some have argued that Verbal IQ (VIQ) (verbal skills) is more negatively affected [10], [21] while others have found only significantly lowered PIQ (visual-spatial skills) [5]. More data would be beneficial for understanding the impact of early lead exposure on specific types of cognitive deficits to further investigate the neurotoxicity of low blood lead on brain function as reflected by cognitive outcomes.

Using a large, community-based, longitudinal cohort sample of Chinese children for whom blood lead concentrations were measured at 3, 4, or 5 years of age, IQ was assessed at 6 years of age, and academic achievement in 3 major subjects (Chinese, Math, and English) was assessed at 8–10 years of age, the present study aims to: 1) assess whether elevated blood lead concentrations are related to preschool children's IQ and later academic achievement in Chinese children; 2) examine whether and the extent to which the relationship between blood lead concentrations and school performance is mediated by IQ; 3) determine which specific components of these aforementioned outcomes are affected to delineate specificity of lead's effect on verbal and performance skills; and 4) identify at what blood lead concentrations <10 µg/dL are reduced cognitive outcomes observed for potential public health implications.

## Participants and Methods

### Subjects

The current study is part of an ongoing longitudinal project, the China Jintan Child Cohort Study, which consists of 1,656 preschool children accounting for 24.3% of all children aged 3–5 years in Jintan city, Jiangsu province, China. Participants were drawn from four preschools chosen to represent the entire city's geographical, social, and economic profiles. Between Fall 2004 and Spring 2005, children aged 3–5 years attending the preschools were invited to participate in this study; signed consent forms were obtained from the parents. Detailed information on this cohort, including subjects, recruitment, and procedures, is reported elsewhere [22]–[25]. Institutional Review Board approval was obtained from the University of Pennsylvania and the ethical committee for research at Jintan Hospital in China.

### Measures

#### Blood lead concentrations in preschool (3–5 years)

Blood specimens were collected only once for each child, when they were 3, 4, or 5 years old, during November 2004 and March 2005 by trained pediatric nurses using a strict research protocol to avoid lead contamination. Samples were frozen and shipped to the Research Center for Environmental Medicine of Children at Shanghai Jiaotong University for the analysis of lead using graphite furnace atomic absorption spectrophotometer [25]–[27]. This laboratory has participated successfully in a CDC-administered quality-control program (Blood Lead Proficiency Testing Program) for the measurement of lead in whole blood. Analysis of each specimen was conducted using a replication procedure, and the mean of the repeated measurements was taken as the final measure. Blood lead reference materials for quality control (QC) were provided by Kaulson Laboratories, New Jersey. QC samples were inserted blindly among the study samples (one QC sample in every 10 study samples. Limit of detection (LOD) of blood lead concentration was 1.8 µg/dL and half of LOD was imputed for 3 (0.2%) samples under LOD, which was among multiple runs (mean LOD).

#### IQ at age 6 years

IQ was assessed by the Chinese version and norms of the Wechsler Preschool and Primary Scale of Intelligence – Revised (WPPSI–R) during children's last year of preschool. The test was constructed by Wechsler [28] to assess the intelligence of children aged 3–7 years and consists of 5 verbal and 5 performance subtests [28]. Verbal subtests are combined to produce a VIQ reflecting verbal skills and crystallized intelligence. Performance subtests combine to produce a PIQ indicative of visual-spatial skills and fluid intelligence. All 10 subtests are combined to produce a Full Scale IQ (FIQ), which is widely recognized as a good measure of general intelligence defined as an average of all cognitive abilities. The Chinese WPPSI was standardized in 1984 and has shown good reliability in Chinese children [29]–[32]. The test was administered by two research assistants trained by a cognitive psychologist. Research assistants who administered the WPPSI were blind to the blood lead concentrations. Children were assessed in a quiet room at their preschool. Detailed procedures and reliability are given in Liu & Lynn [33] and Liu et al. [34].

#### School performance at age 8–10 years

School performance was assessed by standardized city tests on 3 major subjects in Chinese elementary schools: Chinese, Math, and English (as a foreign language). The tests were administered to all children on the same day during the final month of the Fall 2009 semester, when children were in grades 3–5 (aged 8–10 years old). Each test consists mainly of multiple-choice questions and is scored ranging from 0–100. A higher score indicates better performance on the test.

#### Sociodemographic and other Confounding variables

Parents completed a socio-demographic questionnaire to assess family environment at the time of children's IQ testing. Potential confounding variables considered include parent educational and occupational status, father's smoking history and frequency, and mother's smoking during pregnancy. Blood iron was also analyzed at Nanjing Medical University using the same protocol and at the same time as blood lead collection. Whole blood concentrations of iron were determined by atomic absorption spectrophotometry (BH model 5.100 manufactured by Beijing Bohu Innovative Electronic Technology Corporation), with duplicate readings taken with an integration time of two seconds. Further details are provided elsewhere [35].

### Representativeness of Groups

The current study used a sample of 1,341 children (603 girls, 738 boys) for whom blood lead was measured at age 3 years, 4 years, or 5 years, which accounts for 81% of our original cohort. The remaining 19% of the data was either not collected (e.g.: children either moved to other schools or did not respond or refused to participate in follow up) or was unavailable (e.g.: blood samples were not available) for this statistical analysis. Characteristics of the children and their families are summarized in Table 1. There were no significant differences in demographics between children with and without blood lead data [25]. For those children who participated in follow-up for IQ and school performance compared to those who did not participate in follow-up, blood lead concentrations did not differ (t = 1.56; P = 0.120). Meanwhile, complete data on both the IQ and school performance variables were available on 561 subjects. Those with and without complete data were compared on gender, age and grow-place, variables that were available on all subjects at age 6. There was no significant difference between those with complete data and those without complete data on gender, age and grow-place. Therefore, the subjects with complete data are able to represent those without complete data.

**Table 1:** Sample characteristics.

		N‡	%
**Sex**		1341	
	Male	738	55.0
	Female	603	45.0
**Age at blood lead test, Mean (SD)**		1341	4.84(0.86)
	3 years	316	23.6
	4 years	415	30.9
	5 years	610	45.5
**Residence/schools**		1341	
	City (Jianshe)	538	40.1
	Suburban (Huacheng)	521	38.9
	Rural (Xuebu)	282	21.0
**Father's education**		1304	
	≤Middle school	503	38.6
	High school	420	32.2
	College or higher	381	29.2
**Father's occupation**		1262	
	Unemployed	52	4.1
	Physical worker	718	56.9
	Professional worker	492	39.0
**Mother's education**		1305	
	≤Middle school	657	50.3
	High school	384	29.4
	College or higher	264	20.2
**Father smoking**		1273	
	No	563	44.2
	Occasionally	454	35.7
	Several times/wk	41	3.2
	<10 cigarettes/wk	127	10.0
	10–20 cigarettes/wk	71	5.6
	>20 cigarettes/wk	17	1.3
**Iron Status**	Mean (SD)	1341	8.13 (0.83)
**Blood lead (µg/dL)**	Mean (SD)	1341	6.43(2.64)
	<6.0	611	45.6
	6.0 to <8.0	440	32.8
	8.0 to <10.0	185	13.8
	≥10.0	105	7.8

‡ Number of children differs across sample characteristics due to missing values.

### Statistical Analysis

Sample characteristics were summarized by descriptive statistics such as mean, standard deviation (SD), and percentage. To examine the association between blood lead concentration and cognitive function and school performance, we computed bivariate correlations between blood lead concentration and IQ (VIQ, PIQ, and FIQ) and scores on Chinese, Math, and English. We used a nonlinear approach to model the relationship between blood lead concentration and IQ by a locally weighted polynomial regression LOESS model, with estimated 95% confidence band. We identified 8.0 µg/dl, equal to the 80^th^ percentile, as the blood lead concentration at which IQ started to decline. We then divided children into 4 groups according to their blood lead concentration: <6.0 (median), 6.0–8.0 (median to 80^th^ percentile), 8.0–10.0, and ≥10.0 µg/dl. Because the first 2 groups had no significant differences in both IQ and school performance (data available upon request), we merged them and included 3 categories for this report: blood lead concentration <8.0, 8.0 to <10 (8–10), and ≥10 µg/dl.

A series of analysis of variance (ANOVA) were performed to examine the association between different concentrations of blood lead and mean scores of IQ and school performance. General linear models (GLM) were performed to examine the adjusted associations between blood lead concentration and IQ and school performance while controlling for child age at blood lead test, child gender, residence as defined as school location, blood iron level, parent education, parent occupation, and father's smoking. Maternal smoking was not included for analysis as only 3 mothers reported smoking. These demographic variables were selected on the basis of our previous study [25] and/or our preliminary analyses indicating these variables were associated with either or both blood lead concentration and IQ or school performance. Finally, we examined if PIQ mediated the association between blood lead concentration and school performance. We chose PIQ as the potential mediating variable because PIQ is significantly correlated with both the predictor (blood lead concentration) and outcome measure (school performance), and PIQ was measured before school performance data was collected. These two criteria meet the conditions established by Baron and Kenny for potential mediator variable [36]. A p-value<.05 was considered significant. A nonlinear relationship between blood lead concentration and IQ was done using R(2.14.0) loess model. All other analyses were performed using SPSS, Version 17 (Chicago, IL).

## Results

### Sample characteristics

The sample consisted of 603 girls (45.0%) and 738 boys (55.0%), with a mean age of 4.84 years (SD = 0.86) at blood lead testing. Child and family characteristics of the sample are summarized in Table 1. Mean blood lead concentration was 6.43 µg/dL (SD = 2.64). Blood lead concentration was distributed as follows: 7.8% of children were ≥10.0 (N = 105), 13.8% were 8.0–10.0 (N = 185), 32.8% were 6.0–8.0 (N = 440), and 45.6% were <6 µg/dL (N = 611).

### Bivariate correlations between blood lead concentration, IQ, and school performance

Mean IQ and standardized test scores and their bivariate correlations are shown in Table 2. Blood lead concentration was significantly and negatively related to PIQ and scores of Chinese, Math, and English. FIQ was highly correlated with VIQ and PIQ; the correlation between VIQ and PIQ was moderate. Chinese, Math and English were moderately correlated. The correlations between FIQ, VIQ, and PIQ and Chinese, Math, or English were low to moderate.

**Table 2:** Pearson correlations between blood lead concentrations and IQ and school performance.

		Mean (SD)	N‡	Blood lead concentration	VIQ	PIQ	FIQ	Chinese	Math
**Blood lead concentrations (µg/dL)**		6.43(2.64)	1341	1.00					
**IQ**	VIQ	103.95(14.84)	1331	.011	1.00				
	PIQ	104.06(15.07)	1331	−.056*	.498***	1.00			
	FIQ	104.19(14.38)	1331	−.026	.869***	.857***	1.00		
**School Performance**	Chinese	87.87(11.11)	561	−.234***	.241***	.344***	.305***	1.00	
	Math	88.80(11.67)	561	−.200***	.242***	.391***	.375***	.511***	1.00
	English	89.58(13.22)	562	−.207***	.153***	.334***	.294***	.666***	.696***

* p<.05;

** p<.01,

*** p<.001.

‡ Number of children differs across sample characteristics due to missing values.

**Figure 1:**
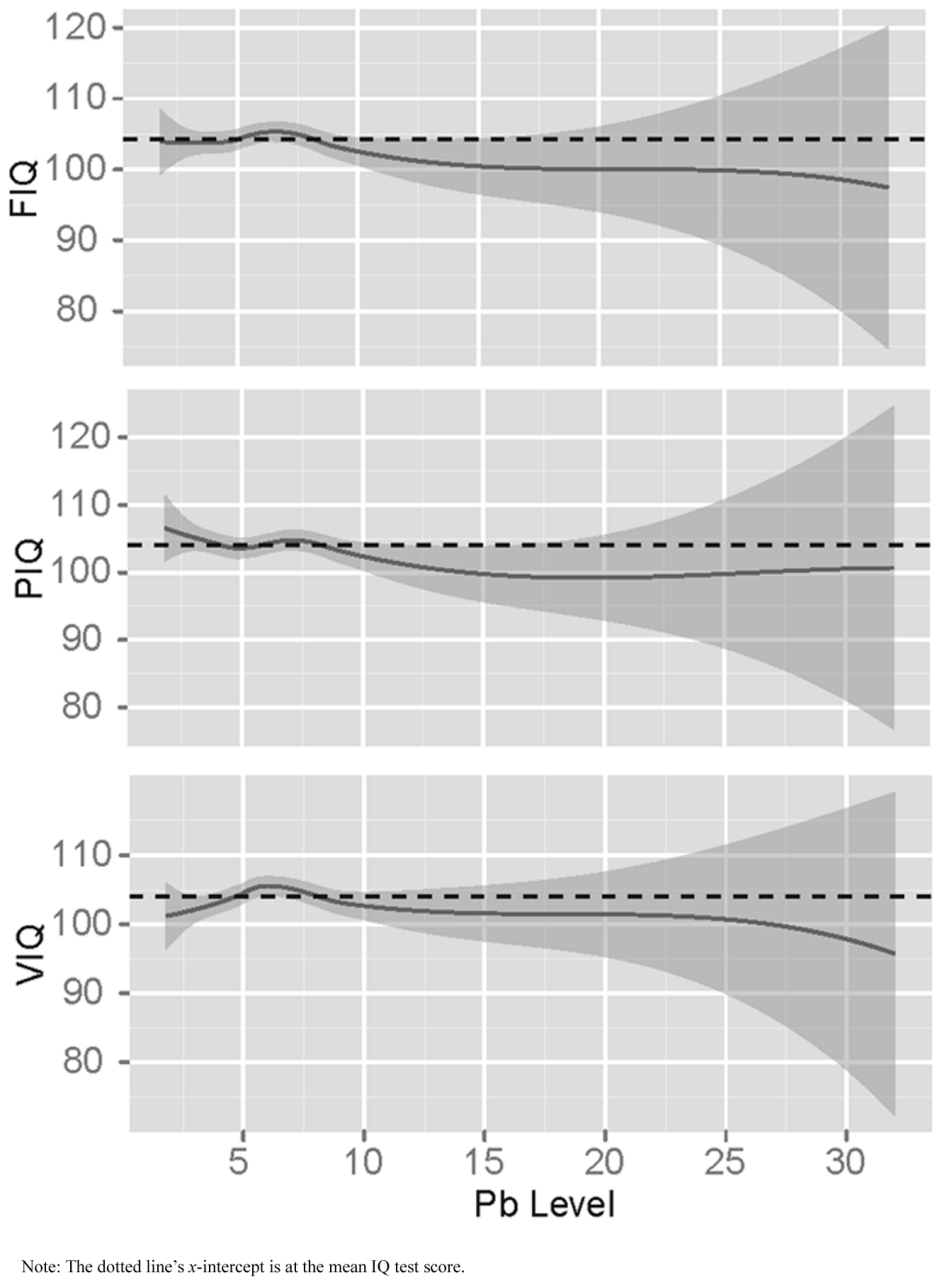
FIQ, VIQ, and PIQ test scores by blood lead concentration (µg/dl) with estimated 95% confidence bands. Note: The dotted line's *y*-intercept is at the mean IQ test score.

The nonlinear relationship between blood lead concentration and FIQ, PIQ, and VIQ, with estimated 95% confidence bands is shown in Figure 1. FIQ started to decline at blood lead concentration 8 µg/dl. Although both PIQ and VIQ declined at blood lead concentration 8 µg/dl, PIQ and VIQ at blood lead concentration <8 µg/dL showed different patterns.

### Differences in IQ and school performance across blood lead concentrations

IQ and school performance means (SD) by 3 categories of blood lead concentration (≥10.0, 8.0–10.0, and <8 µg/dl) is presented in Table 3. All scores declined with increased blood lead concentration. Compared to the blood lead concentration <8 µg/dL group, the blood lead concentration 8.0–10.0 µg/dL group showed a significant 3.71 point PIQ decline and a 2.69 point FIQ decline. The change of VIQ was minor, about 1 point decline.

**Table 3:** Mean IQ and 2009 school performance by blood concentrations of lead in preschool children.

	Blood concentrations of lead (µg/dL)			ANOVA		Post-hoc analysis, LSD (p)		
	<8 (A)	8- <10(B)	≥10 (C)	F	p	A vs. B	A vs. C	B vs. C
**IQ**	N = 1016	N = 182	N = 103					
PIQ	104.46(14.93)	103.32(15.58)	100.75(16.03)	3.04	.048	.349	.018	.168
VIQ	104.23(14.80)	103.65(15.03)	103.08(14.65)	0.36	.696	.627	.453	.755
FIQ	104.55(14.36)	103.66(14.22)	101.86(15.25)	1.78	.169	.442	.072	.313
**School performance**	N = 421	N = 79	N = 49					
Chinese	89.33(9.20)	83.44(13.97)	82.12(16.67)	17.30	.000	.000	<.001	.503
Math	90.32(9.20)	84.29(14.66)	83.04(19.42)	16.32	.000	.000	<.001	.545
English	91.29(10.41)	84.16(18.10)	83.37(19.71)	16.59	.000	.000	<.001	.732

Compared to the blood lead concentration <8 µg/dL group, the blood lead concentration 8.0–10.0 µg/dL group also had significantly lower scores in Chinese, Math, and English. The mean scores significantly declined by 5–6 points in the blood lead concentration 8.0–10.0 µg/dL group compared to blood lead concentration <8 µg/dl. Mean scores did not significantly differ between blood lead concentration 8.0–10.0 and ≥10.0 µg/dL groups.

### Multivariate analysis of blood lead concentration on IQ and school performance

The independent effects of blood lead concentration on IQ and school performance were examined using GLM analyses to adjust for child and family factors. Children with blood lead concentration ≥10.0 µg/dL scored approximately 2 points lower on PIQ and VIQ than those children with blood lead concentration <8 µg/dl (Table 4). However, associations were not significant after adjusting for potential confounders. All Chinese, Math, and English scores significantly declined with elevated blood lead concentration. Math scores declined the most, followed by English and Chinese. Illustratively, compared to children with blood lead concentration <8 µg/dl, those with blood lead concentration 8.0–10.0 µg/dL scored 3–5 points lower on Chinese (β = −3.20, 95%CI = −5.78, −0.63), Math (β = −5.25, 95%CI = −8.14, −2.36), or English (β = −4.33, 95%CI = −7.32, −1.34). Meanwhile those with blood lead concentration ≥10.0 µg/dL scored much lower (Chinese, β = −4.02, 95%CI = −7.11, −0.93; Math, β = −5.27, 95%CI = −8.73, −1,81; English, β = −5.18, 95%CI = −8.76, −1.59) compared to children with blood lead concentration <8 µg/dl.

**Table 4:** Impact of blood concentrations of lead on IQ and school performance in Chinese preschool children (n = 1341).

	Blood concentrations of lead (µg/dL)		
	<8.0	8.0 – <10.0	≥10.0
**IQ (Model 1)**			
**PIQ**	**Ref**	−0.96 (−3.28, 1.36)	−1.90 (−4.89, 1.09)
**VIQ**	**Ref**	−0.48 (−3.36, 2.40)	−1.77 (−4.01, 0.46)
**FIQ**	**Ref**	−1.28 (−4.01, 1.46)	−1.45 (−3.50, 0.67)
**School performance**			
**Chinese: Model 1**	**Ref**	−3.20 (−5.78, −0.63)*	−4.02 (−7.11, −0.93)*
**Model 2**	**Ref**	−2.67 (−5.16, 0.18)*	−3.60 (−6.58, −0.62)*
**Math: Model 1**	**Ref**	−5.25 (−8.14, −2.36)**	−5.27(−8.73, −1,81)**
**Model 2**	**Ref**	−4.46(−7.20, −1.72)**	−4.64(−7.91, −1.36)**
**English: Model 1**	**Ref**	−4.33 (−7.32, −1.34)*	−5.18 (−8.76, −1.59)**
**Model 2**	**Ref**	−3.62 (−6., −0.75)*	−4.62 (−8.05, −1.18)*

* **p<.05,**

** **p<.01.**

Model 1: Adjusting for age at blood lead test, sex, blood iron, school, father's education, mother's education, father's occupation and smoking.

Model 2: Adjusting covariates in model 1 plus PIQ.

### Mediating effect of PIQ on blood lead concentration and school performance

Blood lead concentration and IQ were both significantly correlated with the three school tests, and blood lead concentration was significantly correlated with only PIQ (Table 2). Consequently, it is possible that reduced PIQ could mediate the main effect of blood lead concentration on school performance. This possibility was tested by adding PIQ to Model 1 while adjusting for child, school, and family factors (Table 4). As shown in Model 2, blood lead concentration 8.0–10.0 µg/dL and ≥10.0 µg/dL were still significantly associated with reduced scores on Chinese, Math, and English except for Chinese in children with blood lead concentration 8.0–10.0 µg/dl. Compared with blood lead concentration <8 µg/dL at ages 3–5 years, blood lead concentration ≥10 µg/dL was associated with 3–5 points reduction on school tests at age 8–10 years (Chinese, β = −3.60, 95%CI = −6.58, −0.62; Math, β = −4.64, 95%CI = −7.91, −1.34; English, β = −4.62, 95%CI = −8.05, −1.18). However, the effect as measured by regression coefficient was reduced, indicating that the associations between blood lead concentration and school tests were partially mediated by PIQ.

## Discussion

Lead exposure is an important public health concern, especially in countries that are developing or undergoing rapid economic growth with limited environmental regulations [37]–[39]. However, little is known about the negative impact of low blood lead concentration on later cognitive function in these developing countries, where lead levels may be six-fold higher than in the US [38]–[40]. This study examined the association between blood lead concentration in children at 3–5 years, their IQ at 6 years, and school performance at age 8–10 years. We report several key findings. First, elevated blood lead concentration in early childhood was associated with reduced IQ at age 6 years, particularly for PIQ (visual-spatial skills). Second, even after adjusting for potential confounding variables, including prenatal smoke exposure (exposed to father's smoking) and iron deficiency in childhood, elevated blood lead concentrations were also significantly associated with reduced scores on standardized school tests at age 10 years. Third, IQ partially mediated the blood lead concentration and school performance relationship. Finally and importantly, significant impairments were identified at even 8–10 µg/dL, supporting the view that lead exposure, even <10 µg/dL, is a risk factor for long-term cognitive impairment in children and from a preventative perspective suggests that reduced early childhood lead exposure could promote children's long-term cognitive development and school performance.

Our findings extend previous evidence for an inverse relationship between blood lead concentration and IQ (e.g.: [3], [5]). Our results confirm previous findings by Chandramouli et al. [11] that blood lead concentration <10 µg/dL in early childhood my affect later academic achievement. The present study importantly reports on both children's early IQ and later academic achievement, two different indicators of children's cognitive performance which both have important late-life health and quality-of-life outcomes [41]–[43].

Because of the time gap between IQ and academic achievement assessment and because PIQ was significantly correlated with both blood lead concentration and school performance, we were able to observe a partial mediating effect of PIQ for the blood lead concentration -academic achievement relationship. We hypothesize that lead exposure negatively effects brain growth and development, and that brain alterations are linked to school performance. The exact mechanism between lead exposure and school performance is unclear. Lead is a neurotoxicant, and animal studies suggest that lead exposure may lead to altered brain biochemistry [44], which in turn may result in a disorder of plasticity [45] and learning impairments. Our findings suggest that lead may first alter brain development in children [46]–[49] that subsequently impede further school achievement. This hypothesis is further supported by a functional neuroimaging study demonstrating an inverse correlation between childhood blood lead concentration and activation of the left frontal cortex and middle temporal gyrus, brain regions undergoing rapid development to support language capabilities, during young adulthood [49]. While Yuan et al. also found that dormant circuits in the right hemisphere appeared to be recruited to compensate for these brain deficits circuitry, such a compensatory pathway may not necessarily produce equivalent performance to that achieved by the normative cortical circuitry, and may thus still lead to poorer long-term academic achievement [49].The impairing effects of lead exposure on the brain also extends to adults: structural imaging shows that accumulated occupational lead exposure in adults is associated with changes in cerebral white matter which further affects motor performance [50]. Since PIQ was observed to have a partial mediating effect, our findings also suggest that blood lead concentration may negatively impact other facets of development, such as behavior, that also contribute to reduced school performance. Furthermore, cognitive deficits which were not directly measured, such as poor motivation and self-discipline, may also lead to poor academic achievement [51].

In our sample, blood lead concentration was more strongly associated with PIQ than VIQ. Poorer performance on visual-spatial and visual-motor functioning tests are reported in previous studies of lead exposure [52]–[56]. Notably, by using a large sample size and testing the potential mediating effects of IQ on the blood lead concentration-school performance association, our results extends findings by Jusko et al. [5] that both lifetime average and concurrent blood lead concentration in 6 year old children were significantly associated with lower FIQ and PIQ (p<0.05), but not VIQ (p>0.1). Jusko et al. also suggested that verbal abilities may be more sensitive indicators to cognitive effects of lead exposure during middle-late childhood [5]. In our current study, for the IQ tests, our results showed a significant association between blood lead concentration and only PIQ at age 6 years old, which is almost congruent to children's age at blood lead test, but not VIQ or FIQ. However, for school performance, which was assessed 2–4 years after blood lead, we found significant associations between blood lead concentration and all three tests. These findings suggest that the effect of lead on cognitive deficits appear to be reflected in later childhood (age 8–10 years) through measures of scholastic achievement, which are indicative of verbal working memory and crystallized intelligence [57]. The lack of a significant association between blood lead concentration and VIQ or FIQ at age 6 years could be due to the possibility that IQ is insensitive to brain injury, which in turn is affected by lead exposure [58].

If low concentrations of lead exposure have long-lasting, detrimental cognitive outcomes, a key concern is identifying at what blood lead concentrations <10 µg/dL these outcomes occur. Our results indicate that <8 µg/dL, blood lead concentration was not uniformly associated with decreased outcomes, even after non-linear analysis (Figure 1). IQ, particularly PIQ, noticeably started to decline at ≥8 µg/dL. We also identified significant and pronounced differences between these two groups in later school performance. However, previous studies have reported significant cognitive deficits at blood lead concentration <8 µg/dL in Western children [3]. It could be warranted to examine the cultural and environmental factors associated with the effect of lead on child cognitive development, especially since our study examined Chinese children.

Several limitations in this study should be noted. First, blood lead concentration was measured once for each child from 3–5 years of age. Thus, deficits seen in later childhood may reflect lead exposure at measurement, during the first 2 years of life, or even during the prenatal period. However, while lead susceptibility has been assumed to be greatest during early childhood (e.g., <2 years), several recent studies have shown that blood lead concentrations beyond the age of 2 years are actually more strongly associated with adverse cognitive outcomes, including IQ [3], [7], [59]. Second, blood lead concentrations were measured at different ages within the sample (range of 3–5 years). Nonetheless, even with increasing age, lead concentrations remain fairly constant in early childhood [52]. Third, the effects of low lead exposure on IQ at 10 years were not assessed. However, strong correlations exist in IQ deficits associated with blood lead concentration across childhood and even into adulthood [15], [60]. Still, we are currently collecting a second round of IQ data from this cohort for further analysis. Because children's IQ may not be sensitive enough to detect certain neurocognitive deficits, we are also currently collecting data using more extensive and sensitive neurocognitive tests. Fourth, the present study did not examine other potential confounders, such as the home environment using HOME inventory [5], [61], or additional predictors of neurodevelopmental outcomes, such as parental IQ. While we used parental educational level as a proxy for parental IQ, our findings should nonetheless be replicated in future prospective studies accounting for greater potential confounding factors. Fifth, our findings may also be limited by our high LOD, although only 3 samples (0.2%) were under this LOD. Finally, this study sample was drawn from a region (Jiangsu Province) in China. It is unknown if the findings could be generalized to Chinese children in other regions and children in other countries.

## Conclusion

This study uses a large, Chinese cohort sample to examine the effect of low blood levels in early childhood on specific components of both IQ and school performance. Our findings contribute to the existing evidence regarding low environmental lead exposure and lasting, poor cognitive outcomes. This study also identifies a partial mediating effect of blood lead concentration-IQ-school performance even for lead levels <10 ug/dL. We also examined blood lead concentrations <10 ug/dL using distinct cut-offs and were able to demonstrate that even at blood lead concentration 8–10 ug/dL, children showed significant and persistent poor neurocognitive outcomes. The impact of low-level environmental lead exposure has important implications. Although we focused on school performance as our long-term effect, lead exposure has also been found to affect behavior in children [62], delinquency in adolescence [2], [63], and criminal behavior in adults [17], and has also been shown to effect mental health such as pessimism [64]. It is possible that the negative social and psychological consequences of school failure may drive individuals to negative behavior [65]. Continued follow-up and analysis of multiple measures is necessary to help elucidate both long-term effects and mechanisms through which low lead exposure, particularly lead concentrations below 5 µg/dl (in accordance with the new CDC reference level), in early childhood can lead to adverse neurobehavioral outcomes, which may ultimately result in poor physical and mental well-being. These findings may have important public health implications for early detection and intervention of early childhood lead exposure, especially in Chinese children.
